# Development of Preliminary Candidate Surface Guidelines for Air Force-Relevant Dermal Sensitizers Using New Approach Methodologies

**DOI:** 10.3390/toxics13080660

**Published:** 2025-08-02

**Authors:** Andrew J. Keebaugh, Megan L. Steele, Argel Islas-Robles, Jakeb Phillips, Allison Hilberer, Kayla Cantrell, Yaroslav G. Chushak, David R. Mattie, Rebecca A. Clewell, Elaine A. Merrill

**Affiliations:** 1 AV, Inc., Dayton, OH 45432, USA; 2 711th Human Performance Wing, Air Force Research Laboratory, Wright-Patterson Air Force Base, Dayton, OH 45433, USA; 3 Institute for In Vitro Sciences, Gaithersburg, MD 20878, USA; 4 Henry M. Jackson Foundation for the Advancement of Military Medicine, Inc., Wright-Patterson Air Force Base, Dayton, OH 45433, USA; 5 Eagle Integrated Services, Inc., Wright-Patterson Air Force Base, Dayton, OH 45433, USA; 6 Navy and Marine Corps Force Health Protection Command, Portsmouth, VA 23708, USA

**Keywords:** allergic contact dermatitis, skin sensitization, new approach methodologies, surface guidelines, occupational exposure, in silico modeling, risk assessment

## Abstract

Allergic contact dermatitis (ACD) is an immunologic reaction to a dermal chemical exposure that, once triggered in an individual, will result in an allergic response following subsequent encounters with the allergen. Air Force epidemiological consultations have indicated that aircraft structural maintenance workers may experience ACD at elevated rates compared to other occupations. We aimed to better understand the utility of non-animal testing methods in characterizing the sensitization potential of chemicals used during Air Force operations by evaluating the skin sensitization hazard of Air Force-relevant chemicals using new approach methodologies (NAMs) in a case study. We also evaluated the use of NAM data to develop preliminary candidate surface guidelines (PCSGs, maximum concentrations of chemicals on workplace surfaces to prevent induction of dermal sensitization) for chemicals identified as sensitizers. NAMs for assessing skin sensitization, including in silico models and experimental assays, were leveraged into an integrated approach to predict sensitization hazard for 19 chemicals. Local lymph node assay effective concentration values were predicted from NAM assay data via previously published quantitative models. The derived values were used to calculate PCSGs, which can be used to compare the presence of these chemicals on work surfaces to better understand the risk of Airmen developing ACD from occupational exposures.

## 1. Introduction

Allergic contact dermatitis (ACD) is a Type IV hypersensitivity immune response with lifelong consequences in individuals that have been sensitized to an allergen [1]. Skin sensitization is induced in a susceptible worker through an initial occupational exposure to a chemical substance that binds to skin proteins and initiates a T-cell-mediated immune response [2]. The current consensus is that induction of ACD follows a dose–response model and that a “no expected sensitization induction level” (NESIL) can be derived, wherein the threshold of exposure represents the dose below which induction will not occur [3,4,5]. After the immune system has been primed through induction, even lower levels of dermal exposure to an allergen can elicit a reaction. Thus, ACD can lead to duty-limiting allergies as workers must avoid further contact with the chemical substance to prevent an allergic response.

The United States Air Force (USAF) School of Aerospace Medicine Epidemiology Consult Service Division recently identified potential evidence of occupationally related ACD in USAF personnel performing aircraft structural maintenance tasks [6]. While the presence of dermal sensitizers has been characterized on some work surfaces in USAF aircraft maintenance shops [7], allowable surface levels of potential sensitizers are not commonly available from occupational health organizations. Organizations such as the American Conference of Governmental Industrial Hygienists (ACGIH) have only recently begun developing surface limits, some of which are based on ability of a chemical to induce ACD, that support industrial hygiene evaluation and control [8].

Historically, sensitization hazards have been evaluated using tests in laboratory animals. The local lymph node assay (LLNA), which has been considered a gold standard for identification of skin sensitizers [9], is an in vivo assay conducted in mice that measures a chemical’s ability to induce a proliferation of specialized T-lymphocytes in the lymph nodes following repeated dermal application to the area around the ear [10]. The method uses radioactive labeling of proliferating T cells, as described in the Organisation for Economic Co-operation and Development (OECD) Testing Guideline number 429 [11]. The quantitative result of the test is the three-fold effective concentration (EC3) value, which reflects the concentration at which the proliferation response is sufficiently induced over controls [12]. The EC3 value, which is typically reported as a percentage concentration in the applied substance, is inversely related to sensitizer potency [12]. LLNA EC3 values have traditionally been used to assign Globally Harmonized System (GHS) classification [13], European Center for Ecotoxicology and Toxicology of Chemicals classification [14], and are used as points of departure for quantitative risk assessment [15]. EC3 values can also be used to develop surface limits for occupational exposures to skin sensitizers [16].

More recently, new approach methodologies (NAMs) based on scientific consensus of the biological responses leading to the adverse health effect—or Adverse Outcome Pathways (AOP)—for skin sensitization/ACD have been developed to replace and reduce animal methods [17]. NAM testing incorporates assays that each address different key events in the skin sensitization AOP. For example, the direct peptide reactivity assay (DPRA) assesses the ability of a test chemical to covalently react with cysteine and/or lysine-containing peptides, which represents the first key event of the skin sensitization AOP [18]. A kinetic version of this test (k-DPRA) uses reaction rates with a cysteine-containing peptide to estimate sensitization potency [19]. The cell-based KeratinoSens™ assay addresses keratinocyte activation, the second key event of the AOP. KeratinoSens™ cells are transfected immortalized human keratinocytes (HaCaT) that include a 56-base-pair insertion containing the antioxidant response element (ARE) from the Aldo-Keto Reductase Family 1 Member C2 gene linked to a luciferase reporter. Activation of the signaling pathway with the repressor protein Kelch-like ECH-associated protein 1 (Keap1) and the transcription factor Nuclear factor-erythroid 2-related factor 2 (Nrf2), which binds to the ARE, is a reliable indicator of skin sensitizers in vitro [20,21]. The human cell-line activation test (h-CLAT) characterizes the activation of dendritic cells, addressing the third key event of the AOP. This test measures the upregulation of cluster of differentiation 54 (CD54) and/or cluster of differentiation 86 (CD86) in a THP-1 human monocytic cell line to indicate activation of antigen presenting cells and has been shown to be predictive of sensitizing potential in humans [22].

In silico methods have also been shown to be predictive for identifying potential skin sensitizers [23]. Derek Nexus^®^ (version 6.2.1, Lhasa Limited, Leeds, UK) and the QSAR Toolbox version 4.5 (OECD, Paris, France) are in silico modeling software applications that predict sensitizer potential and EC3 values based on structural similarity of the chemical of interest to those in the database with human and animal test data.

The OECD has developed a guidance document for the use of NAMs for hazard identification and potency prediction [24]. Several possible workflows, or defined approaches, have been explicitly outlined for these purposes that incorporate multiple in chemico, in vitro, and in silico NAMs addressing key events in the skin sensitization AOP. Alternatively, weight of evidence (WoE) approaches that also incorporate in chemico, in vitro, in silico, as well as human and animal data into an integrated assessment can be used to make a determination of sensitization hazard. WoE approaches to determining sensitization hazard have the advantage of allowing inclusion of all available information (human, animal, computational, in chemico, in vitro) and greater flexibility in data interpretation to inform classification and potency determinations [25]. However, WoE approaches rely on expert judgment, which confers a greater level of subjectivity than the OECD-adopted defined approaches.

NAMs have been used to predict EC3 values as a means of evaluating sensitization potency and determining a point of departure (POD) for risk assessment [26]. Five regression models have been proposed, which were derived from an empirical comparison of LLNA EC3 values for ~200 chemicals to specific parameters obtained from the results of the same chemicals in the k-DPRA, KeratinoSens™, and h-CLAT assays and each chemical’s vapor pressure [26]. Each model incorporates a different set of these parameters, with each performing comparably in predicting LLNA EC3 values [26]. The variety of regression models and parameters used in each allows for flexibility in combining this method with OECD defined approaches to efficiently determine hazard, potency, and a POD for potential skin sensitizers [27]. The POD can then be used to derive protective guidance for chemical concentrations on workplace surfaces to prevent induction of skin sensitization in personnel.

This study aimed to better understand the utility of NAMs in characterizing the skin sensitization potential of chemicals used during USAF operations by (1) evaluating the sensitization potential of USAF-relevant chemicals using an integrated approach combining in silico, in chemico, and in vitro NAMs; and (2) developing preliminary candidate surface guidelines (PCSGs, i.e., guidelines for the maximum concentrations of chemicals on workplace surfaces to prevent induction of dermal sensitization) using NAM-derived EC3 values for chemicals identified as sensitizers.

## 2. Materials and Methods

### 2.1. Identification of USAF-Relevant Chemicals and Literature Review

The process to preliminarily identify potential skin sensitizers among USAF-relevant chemicals was previously described by Steele et al. (2021) [28] and Keebaugh et al. (2023) [6] and is briefly summarized here. Researchers compiled candidate chemicals by (1) reviewing Enterprise Environmental, Safety, and Occupational Health Management Information System (EESOH-MIS) usage reports across the USAF; (2) extracting confirmed exposures from USAF occupational field assessments; (3) examining the Occupational Safety and Health Administration (OSHA) expanded standard list tracked at Robins USAF Base; and (4) referencing the five most common allergens reported worldwide [29]. The final chemical candidate list contained 126 substances [28].

As a first pass method for identifying sensitization potential from the literature, the chemical abstract registration number (CASRN) for each chemical on the candidate list was used to search several databases with historical data, including the SkinSensDB database [30], the National Toxicology Program, Interagency Center for the Evaluation of Alternative Toxicological Methods, Integrated Chemical Environment (ICE) database, the OECD eChemPortal, as well as the curated lists of sensitizers maintained by ACGIH, National Institute for Occupational Safety and Health (NIOSH), and OSHA. Eight of the 126 candidate chemicals were excluded from this process because they are gases that were too volatile for testing and were not expected to be present on surfaces. Each chemical was preliminarily designated as a sensitizer, non-sensitizer, or lacking sufficient data to determine sensitization potential based on the information provided by the sources [6].

The chemicals identified as sensitizers that did not have LLNA EC3 values were prioritized for testing in order to develop NAM-based EC3 values to use in the quantitative determination of PCSGs. Chemicals with insufficient literature data that were also predicted to be sensitizers by either in silico model were additionally prioritized so that NAM-based EC3 could be calculated for those that were sensitizers in NAM assays. A few chemicals with LLNA EC3 values were also included for a limited comparison of NAM-based EC3 from this study to existing LLNA EC3. Lastly, chemicals that were anticipated to be difficult to test because of solubility, lipophilicity, or volatility concerns were de-prioritized to arrive at the final list of 19 test chemicals.

### 2.2. In Silico Predictions—Derek Nexus^®^ and QSAR Toolbox

Chemicals were prioritized for testing in this study using Derek Nexus^®^ (version 6.2.1, Lhasa Limited, Leeds, UK) and the QSAR Toolbox version 4.5 (OECD, Paris, France). Chemicals were considered to be potential sensitizers if they were predicted to be sensitizers by either in silico model. Prioritized chemicals were then tested using the NAMs described below.

Derek Nexus^®^ predicted categorical assignments of skin sensitization hazard and EC3 values for each chemical. Predictions were performed using knowledge base “DerekKB 2022 2.0” with species set to mammal and did not account for tautomeric forms of compounds or individual components of mixtures. If a structural alert was indicated at a reasoning level of equivocal, plausible, probable, or certain, then the chemical was considered a sensitizer. Any chemicals that were indicated to be non-sensitizers, or alerts with reasoning levels below equivocal, were considered non-sensitizers. Derek Nexus^®^ also provided predicted EC3 values based on a weighted average of up to 10 nearest neighbor chemicals (as defined by Tanimoto score) with skin sensitization alerts. However, because the validation of the predictivity of the in silico-predicted EC3 values compared to experimentally derived EC3 values was limited [31], in silico-predicted EC3 values were reported and compared to GHS skin sensitization subcategories [13] but were not used in further PCSG calculations.

In QSAR Toolbox, chemicals were analyzed using the automated workflow for skin sensitization. Chemicals were considered sensitizers if they had positive test values in the database or predicted values derived by the software. In some cases, multiple measured values existed for a single chemical, and expert judgment was used to determine whether the measured values could be categorized as “positive” or “negative” based on the species tested (human vs. animal), potency indicated by measured values, and amount of positive vs. negative results. A “positive” determination was conservatively assigned when similar levels of both “positive” and “negative” data were present. In some cases, there were insufficient data available to make a prediction, and/or the chemicals fell outside the domain of applicability of the model. The QSAR Toolbox also predicted EC3 values. However, because no published comparisons of these data with experimentally derived values were found, the predicted EC3 values were not used.

### 2.3. k-DPRA

The k-DPRA calculates the kinetic rates of depletion of a synthetic peptide via the reaction of a given test chemical with cysteine residues of that peptide. All prioritized chemicals were tested in the k-DPRA in accordance with the procedures outlined in OECD Guideline 442C [32]. The maximal kinetic rate [log k_max_ (molar concentration^−1^ seconds^−1^)] of cysteine depletion was used to discriminate GHS subcategory 1A (strong) skin sensitizers from those not categorized as subcategory 1A (other sensitizers or not categorized), with a log k_max_ result of ≥−2.0 used to define those that were GHS 1A. The depletion criteria for the non-kinetic version of the DPRA, the accuracy of which was previously validated against GHS Category 1 for skin sensitizers and LLNA results as described in OECD Guideline 442C [32], were extrapolated to the results to determine positive evidence of skin sensitization potential. Cysteine depletion greater than 13.89% following 24 h incubation with a test chemical at a concentration of 5 millimolar (mM) was considered a positive result.

### 2.4. KeratinoSens™

The KeratinoSens™ assay is designed to evaluate the induction of gene expression via the Nrf2-Keap1 pathway in transfected HaCaT keratinocytes using a luciferase reporter gene. All prioritized chemicals were tested in the KeratinoSens™ assay. The cytotoxicity of the test articles was evaluated based on the nicotinamide adenine dinucleotide phosphate-dependent microsomal enzyme reduction of 3-(4,5-dimethylthiazol-2-yl)-2,5-diphenyl-2H-tetrazolium bromide (MTT) by the KeratinoSens™ cells [33]. The KeratinoSens™ procedures were conducted as described in OECD guideline 442D [34], which is a modification of procedures previously described by Natsch and Emter (2008) and Natsch et al. (2011) [20,35]. The assay was considered positive, and the test article was considered a sensitizer, when there was a significant > 1.5-fold luciferase induction at concentrations lower than 1000 micromolar (µM) and cell viabilities of >70% relative to the vehicle control for at least two valid trials. The accuracy of these criteria was previously validated against GHS Category 1 for skin sensitizers and LLNA results as described in OECD guideline 442D [34]. The maximum fold luciferase induction was reported as I_max_. The concentrations at which 1.5-fold and 3.0-fold luciferase induction occurred were defined as EC_1.5_ and EC_3.0_. The concentrations resulting in 30% and 50% reductions in cell viability were defined as IC_30_ and IC_50_, respectively.

### 2.5. h-CLAT

h-CLAT was conducted as described by OECD Guideline 442E, Annex 1 [36] for chemicals with non-concordant results in the k-DPRA and KeratinoSens™ assays. THP-1 cells were used to evaluate the expression of the cell surface markers CD54 and CD86 (involved in dendritic cell migration) in response to potential sensitizers. Expression of cell surface markers was measured by flow cytometry following cell staining with fluorescein isothiocyanate (FITC) labeled antibodies. Relative fluorescence intensity (RFI) was used as an indicator of CD54 and CD86 expression. Range-finding measurement of cytotoxicity, represented by the concentration resulting in 75% cell viability (CV_75_), was conducted using propidium iodide (PI) staining. RFI was calculated from the Geometric Mean Fluorescence Intensity data acquired by the flow cytometry software (MACSQuantify™ Version 2.10 and FlowLogic 7.2.1). The flow cytometry data acquisition was performed using a MACSQuant Analyzer (Miltenyi Biotec, Bergisch Gladbach, Germany) with a three-laser system capable of both FITC and PI acquisition. A RFI ≥ 200% for CD54 and/or ≥150% for CD86 at cell viabilities ≥ 50% in at least two independent trials was considered a positive result for sensitization. The accuracy of these criteria was previously validated against GHS Category 1 for skin sensitizers and LLNA results as described in OECD guideline 442E, Annex 1 [36]. The minimum induction threshold (MIT) was defined as the lowest of the geometric mean of the test article concentration leading to 200% RFI (EC_200_) for CD54 or 150% RFI (EC_150_) for CD86.

### 2.6. Evaluation of Sensitization Hazard Potential

The results of the NAM assays were used to assign each chemical as “sensitizer” or “non-sensitizer” (i.e., hazard identification). The k-DPRA results were used to identify GHS Subcategory 1A sensitizers as described in Section 2.3 [32]. Additionally, the peptide depletion results (24 h at 5 mM) from the k-DPRA experiment were extrapolated to the DPRA cysteine-only prediction model, and combined with the KeratinoSens™ and h-CLAT results were used to assess sensitization hazard potential analogously to the “2 out of 3” defined approach in OECD Guideline 497 [24]. This testing was performed before the 2024 revisions of OECD Guidelines 442C-E were published [32,34,36], so borderline ranges recommended in that guideline were not considered in the original analysis. However, instances in which inclusion of borderline criteria would affect the interpretation of the assay results are annotated in the data presented below.

### 2.7. Evaluating Sensitizastion Potency and Predicting EC3 Values from Assay Results

EC3 values were predicted from k-DPRA, KeratinoSens™, and h-CLAT results using the methods derived by Natsch and Gerberick [26]. The three assays were used to derive the preliminary three-fold effective concentration, pEC3. The pEC3 is the log of the EC3 value when the EC3 is adjusted to account for molar concentrations used by in vitro testing rather than its standard expression as a per weight basis that is used in the LLNA, as shown in Equation (1).

pEC3 = log (molecular weight/EC3)(1)

Linear transformations were used to convert the logarithmic k-DPRA, KeratinoSens™, and h-CLAT parameters into normalized values spanning a range of zero (no effect) to a positive value of maximal effect. Vapor pressure (VP) in Pascals was also normalized by an equation relating vapor pressure to half-life in the LLNA vehicle [37], with resultant VP_norm_ values below one being set to zero. The normalized parameters were then related to pEC3 using Equations (2)–(4).

pEC3 = 0.42 + 0.40 × log k_max,norm_ + 0.15 × log EC_1.5,norm_ + 0.36 × log IC_50,norm_ − 0.21 × log VP_norm_ (2)

pEC3 = 0.09 + 0.276 × log MIT_,norm_ + 0.22 x EC_1.5,norm_ + 0.34 x log CV_75,norm_ + 0.06 × log IC_50,norm_ − 0.12 × log VP_norm_ (3)

pEC3 = 0.202 + 0.222 × log MIT_,norm_ + 0.40 × EC_3.0,norm_ + 0.313 × log CV_75,norm_ + 0.023 × log IC_50,norm_ − 0.151 × log VP_norm_ (4)

The equations were derived from a multiple linear regression analysis of LLNA-sourced pEC3 values against pEC3 values calculated from normalized k-DPRA, KeratinoSens™, h-CLAT, and VP parameters [26]. Equation (2) uses only k-DPRA and KeratinoSens™ data, whereas Equations (3) and (4) use only KeratinoSens™ and h-CLAT data. The pEC3 values were calculated using equations with only positive assay data so that the most conservative of the pEC3 predictions for each chemical could be determined. Therefore, Equation (2) was used to calculate pEC3 values for chemicals that were positive in k-DPRA and KeratinoSens™ while Equations (3) and (4) were used to calculate pEC3 values for chemicals positive in KeratinoSens™ and h-CLAT. When both Equations (3) and (4) were used, the most conservative pEC3 value of the two results was selected. NAM-predicted EC3 values were compared to GHS skin sensitization subcategories [13].

### 2.8. Calculation of (PCSGs)

PCSGs calculated from EC3s required conversion from percent weight by volume (*w*/*v* %) into a surface concentration applied in the LLNA in units of micrograms per square centimeters (µg/cm^2^). The *w*/*v* % concentration was converted into µg/mL, then multiplied by the amount applied to the ears in the assay (0.025 milliliters [mL] × 2 ears), then divided by mouse ear surface area (2 cm^2^) [16]. PCSGs were derived from the NAM-based PODs by applying a composite adjustment factor to account for uncertainty in translating experimental data into potential human responses and exposure conditions, then converting the PCSGs to units of milligrams per 100 square centimeters (mg/100 cm^2^).

Three adjustment factor values were multiplied together to derive the composite adjustment factor: an adjustment factor for translation to human effects (AF_H_), an adjustment factor for interindividual variability (AF_IV_), and an adjustment factor for exposure considerations (AF_E_). The adjustment factor for interindividual variability was set to ten (AF_IV_ = 10) based on the large human individual variability in induction of sensitization [15]. The AF_E_ represents exposure considerations such as frequency and location of dermal contact in occupational settings, and an AF_E_ = 3 reflects the value recommended for occupational risk assessments [16,38]. A conservative AF_H_ of 10 was used as a default for PODs sourced from the NAM-based EC3. An adjustment factor range of two to 10 has been proposed for translating EC3 values derived from NAM assays into human effect levels [39]. However, the adjustment factor used for a specific chemical depended on the consistency of that value with the evidence of the sensitization potential of that chemical and its close structural analogs [39]. An adjustment factor of six was used instead of ten if close structural analogs of a chemical had comparable LLNA EC3 values. The factor of six was derived from the ratio of mouse LLNA data to human repeat insult patch test data across a wide range of skin sensitization potencies recommended for translating LLNA EC3 data to human effects in occupational risk assessments [16,38].

## 3. Results

### 3.1. In Silico Predictions of Sensitizer Potential

The 22 chemicals preliminarily identified as skin sensitizers in the literature search and 58 chemicals with insufficient literature information to determine their sensitization potential [6] were evaluated using Derek Nexus^®^ (version 6.2.1, Lhasa Limited, Leeds, UK) and the QSAR Toolbox version 4.5 (OECD, Paris, France). This in silico evaluation was used to screen the chemicals for potential sensitizers and prioritize selections for testing with NAM assays. The majority of chemicals with insufficient literature data to preliminarily determine sensitization potential were categorized as non-sensitizers by either in silico model. Eight of the 58 chemicals were predicted to be sensitizers, 45 of the 58 were not predicted to be non-sensitizers, and a conclusive prediction was unable to be made for five chemicals (Table S1). Compounds containing carbonyl functional groups were predominantly identified as sensitizers by the in silico models. A total of 19 chemicals were selected for further testing, including seven that were predicted to be sensitizers by either in silico model and 12 that were identified as sensitizers in the literature review (Table 1). Eight of the chemicals identified as sensitizers in the literature review did not have LLNA EC3 values, while four of the chemicals (xylene, d-limonene, benzaldehyde, and methylglyoxal) did have LLNA EC3s with which to directly compare the NAM-predicted EC3 values.

Derek Nexus^®^ predictions of sensitization potential agreed with 10 of 12 of the chemicals selected for testing that were identified as sensitizers/non-sensitizers in the literature (Table 1). Derek Nexus^®^ predicted EC3 values indicated that many aldehydes, including benzaldehyde, m-tolualdehyde, o-tolualdehyde, valeraldehyde, and propionaldehyde, would be GHS 1A sensitizers. These predictions were primarily based on similarity to dialdehydes, which were much more potent sensitizers than monoaldehydes in the Derek Nexus^®^ database. QSAR Toolbox “predicted” results agreed with the sensitization potential of 6 of 11 of chemicals identified as sensitizers/non-sensitizers in the literature but was unable to make predictions for one chemical identified as a sensitizer in the literature due to lack of data. The literature review agreed with the QSAR Toolbox “measured” results for seven of eight of these chemicals; however, there were four chemicals that were identified as sensitizers in the literature for which insufficient data prohibited an assessment. Some QSAR Toolbox “measured” results disagreed with the QSAR Toolbox “predicted” results. However, as there was limited measured data available for many of these chemicals, the more abundant data on structurally similar chemicals may have weighed more strongly on the predictions than the data for the chemical itself.

### 3.2. NAM Assay Results

**Figure 1:**
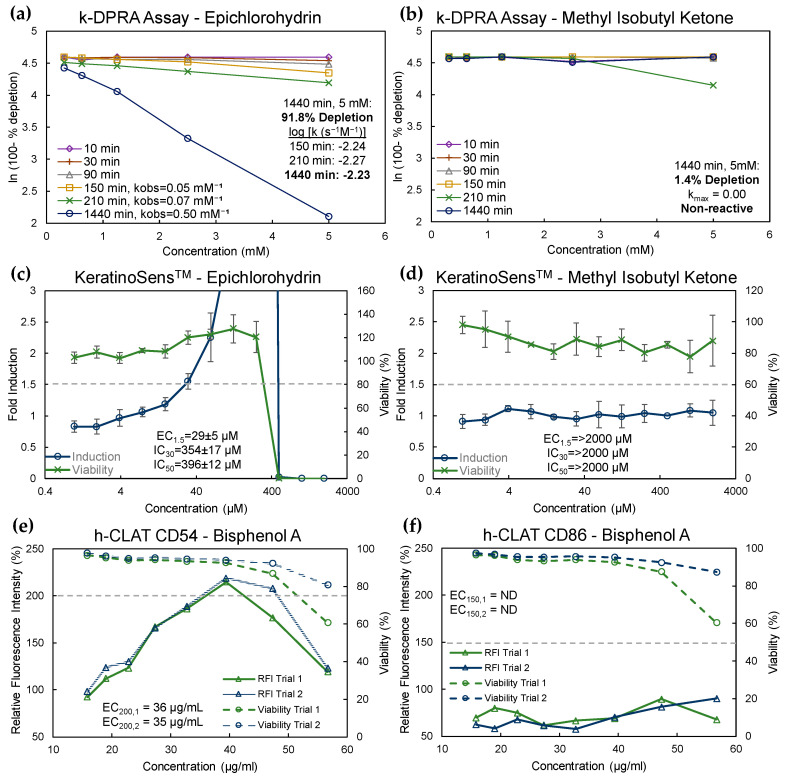
Example assay results for k-DPRA, KeratinoSens™, and h-CLAT. (**a**) k-DPRA results for epichlorohydrin. Data represent the natural logarithm (ln) of the percent of remaining peptide at each concentration in the trial. Where depletion occurs (150, 210, and 1440 min [min]) the observed rate (k_obs_) is calculated in mM^−1^. The logarithm (log) of each k_obs_ divided by the measurement time was then used to determine the maximal depletion rate (log k_max_ = −2.23); (**b**) k-DPRA results for methyl isobutyl ketone, which was non-reactive with peptide depletion of 1.4% at the last measurement timepoint; (**c**) KeratinoSens™ results for epichlorohydrin. Data represent fold luciferase induction (blue line) and cell viability (green line) at each test concentration (mean ± standard deviation of trials). The EC_1.5_, or concentration at 1.5-fold induction (gray dashed line) was 29 ± 5 µM. Reduction in cell viability did not occur until approximately 10-fold higher concentrations; (**d**) KeratinoSens™ results for methyl isobutyl ketone. No increase in luciferase induction or reduction in cell viability occurred at concentrations up to 2000 µM; (**e**) h-CLAT results for CD54 for bisphenol A. Data represent RFI (solid lines) and cell viability (dashed lines) at each test concentration. The EC_200_ (200% RFI represented by gray dashed line) in each trial were 36 µg/mL and 35 µg/mL, respectively. (**f**) h-CLAT results for CD86 for bisphenol A. No increase in RFI above the threshold for positivity (150%, dashed line) was measured.

All 19 chemicals were tested in the KeratinoSens™ assay and k-DPRA. Figure 1a–d shows the results of k-DPRA and KeratinoSens™ testing with two example chemicals: epichlorohydrin and methyl isobutyl ketone. Epichlorohydrin caused 91.8% peptide depletion in the k-DPRA at the 24 h timepoint at a concentration of 5 mM, with a k_max_ of −2.23 (Figure 1a). Minimal peptide depletion (1.4%) resulted when methyl isobutyl ketone was tested in the k-DPRA (Figure 1b), and thus methyl isobutyl ketone was designated as non-reactive based on the criteria for peptide depletion. Epichlorohydrin was positive in the KeratinoSens™ assay, with 1.5-fold induction of ARE genes reached prior to any detectable loss in cell viability (Figure 1c), whereas methyl isobutyl ketone was negative in the assay with no measurable induction (Figure 1d).

Seven of the 19 chemicals (methylglyoxal, 1-naphthylamine, crotonaldehyde, acrylonitrile, epichlorohydrin, acrolein, methacrolein) had peptide depletion that met the criteria to be considered positive (Table S2. Six of these chemicals also had a sufficiently rapid log k_max_ value (log k_max_ ≥ −2) to be designated as GHS Subcategory 1A (“strong”) sensitizers. Only epichlorohydrin did not have a sufficiently rapid log k_max_ for designation as a GHS Subcategory 1A sensitizer out of the chemicals that were positive based on the observed peptide depletion.

A total of 14 of the 19 chemicals were positive in the KeratinoSens™ assay (Table S3). Four of the chemicals (d-limonene, xylene, methyl isobutyl ketone, propionaldehyde) were negative in the assay. Naphthalene was considered an inconclusive result because one trial was negative (I_max_ = 1.30), one trial was positive (I_max_ = 1.73), and the final trial was very close to the threshold for positivity (I_max_ = 1.49). Naphthalene would be considered borderline when considering OECD 442D updated criteria for KeratinoSens™ [34]. p-tert-Butylphenol was considered to be positive in the KeratinoSens™ assay in the original analysis (see Table 1) but would be considered inconclusive (one positive trial and one borderline trial) with the OECD 442E updated criteria [36]. It would have been flagged for an additional trial if those criteria existed at the time. Eight chemicals had non-concordant results between k-DPRA and KeratinoSens™ assays and were subsequently tested in the h-CLAT assay (benzaldehyde, bisphenol A, p-tert-butylphenol, n-phenyl-1-naphthylamine, naphthalene, m-tolualdehyde, valeraldehyde, o-tolualdehyde).

Figure 1e,f show the h-CLAT testing results for bisphenol A. RFI, representative of CD54 and CD86 cell surface marker expression, exceeded the 200% threshold for positivity for CD54 at concentrations that did not cause substantial loss of cell viability in two separate trials (Figure 1e). However, the 150% positivity threshold was not reached for CD86 (Figure 1f). Overall, bisphenol A was considered positive in the h-CLAT assay because at least one marker (CD54) met the positivity criteria in at least two trials. Valeraldehyde, p-tert-butylphenol, m-tolualdehyde, and o-tolualdehyde were also positive in the h-CLAT assay (Table S4). p-tert-Butylphenol, bisphenol A, m-toluealdehyde and o-tolualdehyde would be considered borderline under current OECD 442E criteria for h-CLAT [36]. Benzaldehyde, naphthalene, and n-phenyl-1-naphthylamine were all negative in the h-CLAT assay (Table S4). When considering current OECD 442E criteria, benzaldehyde would be considered inconclusive (based on one negative and one borderline result) and an additional trial would have been prescribed during testing.

### 3.3. Assessment of Sensitization Hazard and Potency

Assay-predicted EC3 values for 1-naphthylamine, crotonaldehyde, acrolein, bisphenol A and methacrolein categorized all five chemicals as GHS Subcategory 1A strong sensitizers (Table 2). Positive results in two NAM assays also indicated that all five chemicals were sensitizers. The evidence was also clear that seven other chemicals were sensitizers (acrylonitrile, epichlorohydrin, methylglyoxal, p-tert-butylphenol, m-tolualdehyde, valeraldehyde, and o-toluladehyde). Each chemical was positive in at least two of the three tested NAM assays and categorized as GHS Subcategory 1B by the assay-predicted EC3 value. Xylene, d-limonene, methyl isobutyl ketone, benzaldehyde, and propionaldehyde were determined to be non-sensitizers based on negative peptide depletion and KeratinoSens™ results. Naphthalene was considered to be a non-sensitizer based on negative peptide depletion and h-CLAT results.

N-phenyl-1-naphthylamine was assigned inconclusive for sensitization hazard because of equivocal results from the three assays (Table 2). Although the h-CLAT assay result was negative, n-phenyl-1-naphthylamine is highly lipophilic with an octanol-water partition coefficient (log K_ow_) of 4.2 [40]. Compounds with log K_ow_ of >3.5 have the potential for false negative responses in the h-CLAT assay [36], so the h-CLAT results for n-phenyl-1-naphthylamine were considered to be inconclusive.

### 3.4. Preliminary Candidate Surface Guidelines

PCSGs in mg/100 cm^2^ were calculated from assay-derived EC3 values for the 12 chemicals determined to be sensitizers based on the NAM assays (Table 3). A multiple of adjustment factors equaling 300 (AF_IV_ = 10, AF_E_ = 3, AF_H_ = 10) was used as the default adjustment factor to account for uncertainty in translating experimental data into human responses and actual exposure conditions with most chemicals. Valeraldehyde and methylglyoxal were given lower AF_H_ adjustment factors of six based on the presence of close structural analogs in the Derek Nexus^®^ model. Valeraldehyde had three close structural analogs (Tanimoto scores: 0.7–0.8) identified by Derek Nexus^®^; all three were also very weak sensitizers with an average LLNA EC3 value of 61%. This suggested the prediction of 16% for valeraldehyde was sufficiently conservative and justified a lower AF_H_ adjustment factor and composite adjustment factor of 180 (AF_IV_ = 10, AF_E_ = 3, AF_H_ = 6). Similarly, methylglyoxal, with a NAM-predicted EC3 of 3.6%, had three close structural analogs (Tanimoto scores: 0.7–0.9) identified by Derek Nexus^®^ with an average LLNA EC3 value of 16%, suggesting a lower composite adjustment factor of 180 (AF_IV_ = 10, AF_E_ = 3, AF_H_ = 6) was warranted (the LLNA EC3 of methylglyoxal was ignored for this analysis to evaluate the NAM-based methods).

## 4. Discussion

### 4.1. Comparison of NAM-Based Sensitization Hazard Potential to In Vivo Evidence from the Literature

The information available from literature for chemicals tested in this study was generally consistent with the assignments of sensitization potential determined from NAMs. Bisphenol A has positive human patch-test studies and a mouse ear-swelling test [41] that support its assignment as a sensitizer. The assignment of p-tert-butylphenol as a sensitizer was also supported by the literature. However, it should be noted that p-tert-butylphenol would be inconclusive using NAMs if applying the current OECD 497 criteria to the existing dataset (inconclusive KeratinoSens™ and borderline h-CLAT). Although p-tert-butylphenol was negative in the guinea pig maximization test (GPMT), there are data on positive reactions to p-tert-butylphenol in human diagnostic patch testing [42]. Epichlorohydrin has been shown to have sensitization potential in both guinea pig [43] and human testing [44], which is consistent with the NAM-based assignment of epichlorohydrin as a sensitizer. Other chemicals preliminarily identified as sensitizers from the initial literature review including methylglyoxal, 1-naphthylamine, crotonaldehyde, acrylonitrile, were also clearly identified as sensitizers by k-DPRA and KeratinoSens™ testing. Xylene had a very weak LLNA EC3 value of 96% but was effectively defined in the literature as a non-sensitizer, which aligns with the NAM-based assignment.

There were two cases in which the NAM-based assignment and literature review-based assignment of sensitization potential did not align where the animal data and human data also disagreed. D-limonene was preliminarily identified as a sensitizer in the literature and had a weak median LLNA EC3 value of 30%, which would appear to conflict with its assignment as a non-sensitizer in the NAMs tested in this study. However, a recent analysis of d-limonene as a human skin sensitizer that incorporated animal, human, and in vitro data determined that the weight of evidence did not support its classification as a human sensitizer despite positive LLNA results [25]. The stated rationale was that d-limonene was a very weak sensitizer in human tests, and it was suggested that the stronger results of animal testing may have been false positives resulting from the presence of skin irritation associated with high levels of d-limonene exposure. There is also evidence from guinea pig testing that d-limonene is not itself sensitizing, but is a pre-hapten, and the oxidation product from the aging of d-limonene in air over weeks to months is the active substance inducing sensitization [45]. Therefore, the dependence of sensitization potential on the level of aging of d-limonene is a potential limitation to both NAMs and animal assays.

The assignment of benzaldehyde as a non-sensitizer from the NAMs disagreed with human patch testing data that indicates it is a sensitizer, albeit a weak sensitizer [25]. If borderline ranges in the OECD 442E updated criteria are applied, the h-CLAT result with existing trials would be considered inconclusive (with the existing trials being negative and borderline) and an additional repeat of the h-CLAT assay would have been recommended during testing. The only overall outcome of the h-CLAT for benzaldehyde, however, would have been either negative (if a hypothetical final trial was negative) or borderline (if the hypothetical final trial was positive or borderline), so a positive determination of benzaldehyde as a sensitizer would not have been considered because the only positive assay would have been KeratinoSens™. Benzaldehyde was not sensitizing at the highest concentration tested in the LLNA (25%) [25], which is consistent with results from the NAMs, but also inconsistent with human data where it was sensitizing at lower concentrations. Although the LLNA was used as a comparison benchmark for the NAM-based assays in deriving the EC3 prediction used in this study, the cases demonstrate that results from animal testing are not always representative of effects in humans.

Although n-phenyl-1-naphthylamine was assigned as inconclusive based on NAM data, it produced a strong positive response in the GPMT [46]. There is also one human case study reporting ACD associated with exposure to n-phenyl-1-naphthylamine [47]. The potential for a false negative result because n-phenyl-1-naphthylamine is highly lipophilic is one possible explanation of the discrepancy between NAMs and in vivo data for that chemical, as the h-CLAT test has been shown to produce false negative results for chemicals with a log K_ow_ of >3.5 [48]. In addition, n-phenyl-1-naphthylamine contains an aromatic amine, a functional group that may be under-predicted in the k-DPRA due to its requirement for oxidative conditions to be correctly identified as a sensitizer [32]. This limitation may also explain its classification as a non-sensitizer based on peptide depletion.

The findings of the present study were compared to similar NAM-based testing in the literature. The negative h-CLAT results in this study disagreed with published positive h-CLAT results for benzaldehyde; however, it was unclear if borderline ranges were considered in those results [25]. d-Limonene was negative in both the KeratinoSens™ assay and peptide depletion during the present study but had mixed results in the literature [25,49]. While the reasons for the variability in results for these chemicals would require further investigation, this would provide a better understanding of intra- and inter-laboratory reproducibility that is a key factor in supporting the validity and acceptability of NAMs for risk assessment without relying on comparisons to animal data [50]. The results of this work are supported by a previous report that evaluated chemicals commonly found in wearable devices that applied the same NAM-derived EC3 strategy to characterize skin sensitization risks associated with this class of products [51]. The report confirmed skin sensitization hazards, resolved inconsistencies in animal studies and consumer reports, and established a POD for next-generation risk assessment.

### 4.2. Calculating PCSGs from NAM-Derived EC3 Values

The composite adjustment factors of 180 to 300 used in this study fell within the wide range of composite adjustment factors used for skin sensitization quantitative risk assessments (3–10,000, although up to 300 is typically used in practice) [38]. The composite adjustment factor range for NAM-based EC3 values used during this study (180–300) was slightly higher than composite adjustment factors of 50–180 that have been recommended specifically for surface guidelines for occupational exposures from LLNA EC3 values [16,38].

An AF_H_ of six was used when a chemical had sufficiently close structural analogs, which was based on the recommendation for translating LLNA EC3 values to human effects for occupational exposures [16,38]. Some have argued for an interspecies adjustment factor of 15 based on the 95th percentile of probability distributions of the LLNA to human data ratios [52], whereas the strong correlation of LLNA data to human data suggests that there is not a routine need for an AF_H_ [53]. It is also common to use no AF_H_ in fragrance ingredient risk assessments [15]. Therefore, the use of the AF_H_ of six in the present study was considered a reasonably conservative compromise among the range of possibilities and aligned with the occupational literature.

An AF_IV_ of 10 was used to account for interindividual variability in humans. Naumann and Arnold (2019) [16] did not define an AF_IV_; however, several other quantitative risk assessments did apply an AF_IV_ uncertainty factor for dermal sensitization [38]. Although an adjustment factor for vehicle/matrix effects on dermal penetration of the chemical of interest is sometimes used [16], it was not applied within the current effort because even in consumer products with direct application to the skin an adjustment factor of one is recommended unless the product contains known penetration enhancers [15].

Recommendations for dermal sensitization risk assessments for fragrance materials sometimes incorporate both an adjustment factor of three for repeated use of a product, and another factor of three for use on body areas, such as the hands, that are more prone to inflammation [15]. While both scenarios may apply to the occupational setting, an AF_E_ of three was used to represent exposure considerations as defined in previous occupational risk assessments [16,38] which accounted for differences between intentional dermal application in cosmetics and accidental exposure in occupational settings [16].

### 4.3. Limitations of NAM-Based Methods for Developing PCSGs

The focus of the present study was to identify PODs for quantitative risk assessment (as a part of development of PCSGs), and thus k-DPRA, KeratinoSens, and h-CLAT were used to generate data to use with the Natsch and Gerberick (2022) regression models for EC3 prediction [26]. k-DPRA and KeratinoSens™ were initially tested because they had the best concordance with the in vivo EC3 and were higher throughput. While the incorporation of DPRA would have removed the need for extrapolation and increased confidence in sensitization hazard predictions, the use of k-DPRA still provides relevant information that supports hazard assessment at the same timepoint and concentration. Likewise, the integrated testing strategies, ITSv1 and ITSv2 in OECD Guideline 497 could have been used to assign sensitization hazard sub-categorization by integrating extrapolated peptide depletion and h-CLAT data with in silico model results; however, there were only h-CLAT data for some of the chemicals tested when the results between the k-DPRA and KeratinoSens™ were not concordant, and there were limitations with the in silico modeling described below.

There were several limitations identified when developing PCSGs using this relatively new approach. The derivations of Equations (2)–(4) by Natsch and Gerberick (2022) [26] were performed using existing LLNA and NAM data from the literature and an OECD database. Much of the published research on dermal sensitization data has been generated for cosmetic and consumer products, and the chemicals used in that research are potentially structurally distinct from chemicals used by the USAF in occupational scenarios. Therefore, the applicability of NAM-predicted EC3 values that were developed for consumer products to AF-relevant chemicals is yet to be fully validated. The lack of overlap in chemical space is evidenced by the limited LLNA EC3 values available for comparison in this study. In particular, aldehydes are one class of chemicals studied that would benefit from additional LLNA EC3 data with which to compare NAM-derived EC3 values. A previous study noted that NAM-derived EC3 values were least predictive of aldehyde LLNA EC3 values [37]. This limited predictivity is potentially a result of poor reactivity of aldehydes in the k-DPRA because the conditions of the assay are not favorable for Schiff base formation [37].

Aldehydes (excluding α,β-unsaturated aldehydes) were also a chemical class for which Derek Nexus^®^ appeared to potentially overpredict potency in the chemicals that were studied. Benzaldehyde was predicted to be a much stronger sensitizer than was indicated in the literature, and several aldehydes were predicted to be substantially stronger sensitizers than the NAM-derived EC3 values. One potential explanation for the discrepancy is that Derek Nexus^®^ included several dialdehydes as close structural matches for the aldehydes of interest. These dialdehydes were listed as very strong sensitizers in the Derek Nexus^®^ database, which may have weighted the overall prediction in that direction for the aldehydes in this study. The increased peptide reactivity of dialdehydes compared to monoaldehydes has been demonstrated experimentally with glutardialdehyde being 170 times more reactive compared to the corresponding monoaldehyde valeraldehyde [54]. This substantial difference in reactivity would justify the manual exclusion of dialdehydes from aldehyde predictions [55]. Excluding dialdehydes for the chemicals of interest in the present study brings the predictions from the GHS Subcategory 1A level to the GHS Subcategory 1B level that more closely agreed with assay predictions.

The EC3 prediction regression equations used in this study may mildly underpredict the potency of stronger sensitizers due to a limited dynamic range in the potency assessment, as has been suggested previously [27]. This may explain why methylglyoxal was predicted to be a GHS Subcategory 1B sensitizer using the NAM-derived EC3 despite being a GHS Subcategory 1A sensitizer based on its LLNA EC3. It could also explain the discrepancy between methylglyoxal and acrylonitrile being predicted to be GHS Subcategory 1A sensitizers by the k-DPRA results, while only being predicted as GHS Subcategory 1B using the EC3 regression-based method.

## 5. Conclusions

NAM-based assays were used to evaluate the skin sensitization hazard potential of 19 chemicals of relevance to the USAF in a small case study. PCSGs were then derived for 12 of these chemicals that were predicted to be sensitizers. The derived PCSGs can be used for comparison to concentrations on work surfaces to better understand the risk of Airmen developing ACD from occupational exposures. Wipe samples of a known area (e.g., 100 cm^2^) can be collected from high-contact surfaces in occupational settings for processes that are known to use a given chemical, and the total amount of that chemical present on the wipe can be determined using analytical methods. The mass of chemical collected on the wipe can then be divided by the area wiped to give the surface concentration of the chemical in µg/cm^2^ or mg/100 cm^2^. If the surface concentration of that chemical is higher than the PCSG, then there would potentially be elevated risk for induction of ACD.

The favorable comparison of NAM-based assessments with known sensitizers and LLNA EC3 values supports the use of these methods for developing PCSGs for chemicals without surface limits to improve force health protection and reduce potential occupational risk from skin sensitizing chemicals. Because of the potential difference between USAF-relevant chemicals and the chemicals used to develop the predictive models, further evaluation of the applicability domain for these assays and EC3 predictions is needed. Nonetheless, the sensitizing potential of the occupational chemicals in this study was generally well-predicted by the NAM-based methods and the PCSGs provide a reasonable starting point for developing workplace risk mitigation strategies. This finding is also consistent with a recent study that used NAM-based EC3 methods to evaluate skin sensitization in chemicals in wearable devices. These studies provide practical examples of the utility of non-animal toxicological methods in increasing the efficiency of risk assessment compared to traditional animal testing to support Force health protection in the USAF.

## Figures and Tables

**Table 1:** In silico results for chemicals prioritized for further testing with NAM assays. The Derek Nexus^®^ output designated whether a chemical was predicted to be a sensitizer or non-sensitizer along with an associated level of confidence for that prediction (e.g., ^1^ probable, ^2^ plausible, ^3^ equivocal). Chemicals were also assigned based on OECD Toolbox measured/predicted results. ^4^ Multiple data were available in the QSAR Toolbox database, expert judgment applied and sensitizer assignment was designated as a conservative measure. “ND”: no result was returned by the model due to insufficient data for that chemical. GHS Subcategories—1A: strong sensitizer, EC3 ≤ 2%; 1B: other sensitizer, EC3 > 2%.

CASRN	Chemical Name	Preliminary Assignment from Literature Review	Derek Nexus^®^ Predicted Assignment	Derek Nexus^®^ Predicted EC3	QSAR Toolbox Measured Assignment	QSAR Toolbox Predicted Assignment
5989-27-5	d-Limonene	Sensitizer	Sensitizer ^1^	8.7% (1B)	Sensitizer	Sensitizer
1330-20-7	Xylene	Non-Sensitizer	Non-Sensitizer	Non-Sensitizer	Non-Sensitizer	Non-Sensitizer
108-10-1	Methyl Isobutyl Ketone	Insufficient Data	Non-Sensitizer	Non-Sensitizer	Non-Sensitizer	Sensitizer
123-38-6	Propionaldehyde	Insufficient Data	Sensitizer ^2^	0.19% (1A)	Non-Sensitizer	Sensitizer
100-52-7	Benzaldehyde	Sensitizer	Sensitizer ^3^	0.04% (1A)	Sensitizer ^4^	Non-Sensitizer
91-20-3	Naphthalene	Sensitizer	Non-Sensitizer	Non-Sensitizer	ND	Sensitizer
90-30-2	n-Phenyl-1-naphthylamine	Sensitizer	Non-Sensitizer	Non-Sensitizer	Sensitizer	Non-Sensitizer
78-98-8	Methylglyoxal	Sensitizer	Sensitizer ^2^	4.8% (1B)	ND	Non-Sensitizer
134-32-7	1-Naphthylamine	Sensitizer	Sensitizer ^3^	35% (1B)	Non-Sensitizer	ND
4170-30-3	Crotonaldehyde	Sensitizer	Sensitizer ^2^	2.4% (1B)	ND	Sensitizer
107-13-1	Acrylonitrile	Sensitizer	Sensitizer ^1^	ND	Sensitizer	Sensitizer
106-89-8	Epichlorohydrin	Sensitizer	Sensitizer ^2^	1.1% (1A)	ND	Sensitizer
107-02-8	Acrolein	Insufficient Data	Sensitizer ^2^	1.9% (1A)	ND	Sensitizer
78-85-3	Methacrolein	Insufficient Data	Sensitizer ^2^	2.7% (1B)	ND	Sensitizer
80-05-7	Bisphenol A	Sensitizer	Sensitizer ^3^	5.6% (1B)	Sensitizer	Non-Sensitizer
98-54-4	p-tert-Butylphenol	Sensitizer	Sensitizer ^3^	3.5% (1B)	Sensitizer ^4^	Non-Sensitizer
620-23-5	m-Tolualdehyde	Insufficient Data	Sensitizer ^3^	0.05% (1A)	ND	Sensitizer
110-62-3	Valeraldehyde	Insufficient Data	Sensitizer ^2^	0.26% (1A)	ND	Sensitizer
529-20-4	o-Tolualdehyde	Insufficient Data	Sensitizer ^3^	0.05% (1A)	ND	ND

**Table 2:** Assessment of sensitization hazard potential based on NAM assays. Assessment of each chemical as a sensitizer/non-sensitizer was made based on NAM-predicted EC3 values and associated GHS Subcategories (1A: strong sensitizer, EC3 ≤ 2%; 1B: other sensitizer, EC3 > 2%), k-DPRA-predicted GHS Subcategory (1A or non-1A), and positivity in two of the three NAM assays: peptide depletion in k-DPRA at 24 h and 5 mM, KeratinoSens™, and h-CLAT. ^a^ Inconclusive and ^b^ borderline outcomes when considering OECD 442D (KeratinoSens™) and OECD 442E (h-CLAT) updated criteria. ^c^ EC3 values derived from both Equations (3) and (4) are listed, the more conservative (lower) value was used for GHS subcategory determination and PCSG calculation. N/A: not applicable.

Chemical Name	Literature LLNA EC3	k-DPRA Peptide Depletion (%)	k-DPRA GHS Subcategory Prediction	KeratinoSens™ Results	h-CLAT Results	NAM-Based EC3 (GHS SubCat)	Determination
d-Limonene	30% (1B)	1.0	Non-GHS 1A	Negative	N/A	N/A	Non-Sensitizer
Xylene	96% (1B)	1.0	Non-GHS 1A	Negative	N/A	N/A	Non-Sensitizer
Methyl Isobutyl Ketone	N/A	1.4	Non-GHS 1A	Negative	N/A	N/A	Non-Sensitizer
Propionaldehyde	N/A	1.0	Non-GHS 1A	Negative	N/A	N/A	Non-Sensitizer
Benzaldehyde	>25% (1B)	4.6	Non-GHS 1A	Positive	Negative ^a^	N/A	Non-Sensitizer
Naphthalene	N/A	7.3	Non-GHS 1A	Inconclusive ^b^	Negative	N/A	Non-Sensitizer
n-Phenyl-1-naphthylamine	N/A	4.0	Non-GHS 1A	Positive	Inconclusive	N/A	Inconclusive
Methylglyoxal	0.42% (1A)	39.2	GHS 1A	Positive	N/A	3.6% (1B)	Sensitizer
1-Naphthylamine	N/A	21.0	GHS 1A	Positive	N/A	1.5% (1A)	Sensitizer
Crotonaldehyde	N/A	91.9	GHS 1A	Positive	N/A	0.37% (1A)	Sensitizer
Acrylonitrile	N/A	99.0	GHS 1A	Positive	N/A	7.7% (1B)	Sensitizer
Epichlorohydrin	N/A	91.8	Non-GHS 1A	Positive	N/A	7.7% (1B)	Sensitizer
Acrolein	N/A	99.0	GHS 1A	Positive	N/A	0.29% (1A)	Sensitizer
Methacrolein	N/A	96.0	GHS 1A	Positive	N/A	0.47% (1A)	Sensitizer
Bisphenol A	N/A	2.2	Non-GHS 1A	Positive	Positive ^b^	1.5%, 1.6% (1A)	Sensitizer
p-tert-Butylphenol	N/A	1.0	Non-GHS 1A	Positive ^a^	Positive ^b^	3.3%, 7.9% (1B) ^c^	Sensitizer
m-Tolualdehyde	N/A	1.0	Non-GHS 1A	Positive	Positive ^b^	5.2%, 7.6% (1B) ^c^	Sensitizer
Valeraldehyde	N/A	1.0	Non-GHS 1A	Positive	Positive	16%, 15.5% (1B) ^c^	Sensitizer
o-Tolualdehyde	N/A	1.0	Non-GHS 1A	Positive	Positive ^b^	5.4%, 7.7% (1B) ^c^	Sensitizer

**Table 3:** Preliminary candidate surface guidelines. PCSGs were calculated from the POD based on NAM assay-derived EC3 values for chemicals predicted to be sensitizers. A composite adjustment factor was applied to each value to account for potential uncertainty and variability in the calculation of the PCSGs.

Chemical Name	NAM POD (µg/cm^2^)	Composite Adjustment Factor	PCSG (mg/100 cm^2^)
Methylglyoxal	900	180	0.5
Bisphenol A	380	300	0.1
p-tert-Butylphenol	830	300	0.3
1-Naphthylamine	380	300	0.1
Crotonaldehyde	93	300	0.03
Acrylonitrile	1900	300	0.6
Epichlorohydrin	1900	300	0.6
Acrolein	73	300	0.02
Methacrolein	120	300	0.04
m-Tolualdehyde	1300	300	0.4
Valeraldehyde	4000	180	2
o-Tolualdehyde	1400	300	0.5

## Data Availability

The data presented in this study are available on request from the corresponding author due to public release restrictions by the United States Air Force and Department of Defense.

## Supplementary Materials

The following supporting information can be downloaded at: https://www.mdpi.com/article/10.3390/toxics13080660/s1, Table S1: Chemical prioritization; Table S2: k-DPRA data; Table S3: KeratinoSens™ data; Table S4: h-CLAT data.

## Disclaimer

Distribution Statement A. Approved for public release. Distribution is unlimited. Case Number AFRL-2025–1483, 27 March 2025 and Case Number AFRL-2025-3630, 24 July 2025. The views, opinions, and/or findings expressed in this article are those of the authors and do not reflect the official guidance or position of the United States Government, the Department of Defense, the United States Air Force or the United States Space Force. The content or appearance of hyperlinks does not reflect an official DoD, Air Force, Air Force Research Laboratory position or endorsement of the external websites, or the information, products, or services contained therein. The views expressed do not reflect the official guidance or position of AV, Inc., Henry M. Jackson Foundation for the Advancement of Military Medicine, Inc., or Eagle Integrated Services.

## Abbreviations

The following abbreviations are used in this manuscript:

ACD	Allergic contact dermatitis
ACGIH	American Conference of Governmental Industrial Hygienists
AF_E_	Adjustment factor for exposure considerations
AF_H_	Adjustment factor for translation to human effects
AF_IV_	Adjustment factor for interindividual variability
AOP	Adverse outcome pathway
ARE	Antioxidant response element
CASRN	Chemical Abstract Service Registry Number
CD54	Cluster of differentiation 54
CD86	Cluster of differentiation 86
cm^2^	Square centimeters
CV_75_	75% cell viability
DPRA	Direct Peptide Reactivity Assay
EC1.5	Concentration at 1.5-fold luciferase induction in KeratinoSens™
EC150	Concentration at 150% relative fluorescence intensity in human cell line activation test
EC200	Concentration at 200% relative fluorescence intensity in human cell line activation test
EC3	Three-fold effective concentration in local lymph node assay
EC3.0	Concentration at 3.0-fold luciferase induction in KeratinoSens™
EESOH-MIS	Enterprise Environmental, Safety, and Occupational Health Management Information System
FITC	fluorescein isothiocyanate
GHS	Globally Harmonized System
GPMT	Guinea pig maximization test
HaCaT	Transfected immortalized human keratinocytes
h-CLAT	Human cell line activation test
IC_30_	Concentration resulting in 30% reduction in cell viability in KeratinoSens™
IC_50_	Concentration resulting in 50% reduction in cell viability in KeratinoSens™
I_max_	Maximum fold luciferase induction in KeratinoSens™
k-DPRA	Kinetic direct peptide reactivity assay
Keap1	Kelch-like ECH-associated protein 1
k_max_	Maximal rate of depletion in the kinetic direct peptide reactivity assay
LLNA	Local lymph node assay
mg/100 cm^2^	Milligrams per 100 square centimeters
mL	Milliliters
mM	Millimolar
MTT	3-(4,5-dimethylthiazol-2-yl)-2,5-diphenyl-2H-tetrazolium bromide
NAM	New approach methodology
NESIL	No expected sensitization induction level
NIOSH	National Institute for Occupational Safety and Health
Nrf2	Nuclear factor-erythroid 2-related factor 2
OECD	Organisation for Economic Co-operation and Development
OSHA	Occupational Safety and Health Administration
PCSG	Preliminary candidate surface guideline
pEC3	Preliminary three-fold effective concentration
PI	propidium iodide
POD	Point of departure
QSAR	Quantitative structure-activity relationship
RFI	Relative fluorescence intensity
USAF	United States Air Force
VP	Vapor pressure
*w*/*v* %	Percent weight by volume
WoE	Weight of evidence
µg/100 cm^2^	Micrograms per 100 square centimeters
µg/mL	Micrograms per milliliter
µM	Micromolar
